# Heterogeneity in internal medicine chief residency selection processes and role preparation: an opportunity for improvement

**DOI:** 10.1186/s12909-025-07496-x

**Published:** 2025-07-02

**Authors:** Benjamin L. H. Jones, Lenny López, Megha Garg

**Affiliations:** 1 Department of Medicine, University of California Los Angeles, 757 Westwood Plaza, Suite 7236, Los Angeles, CA 90095 USA; 2 Department of Medicine, University of California San Francisco, San Francisco, CA USA; 3 San Francisco Veterans Affairs Medical Center, San Francisco, CA USA

**Keywords:** Graduate medical education, Chief residency, Selection

## Abstract

**Background:**

The chief resident (CR) role is integral to graduate medical education and the administrative, educational, and clinical functions of residency programs. There are limited data on the selection and job training of CRs in internal medicine (IM).

**Methods:**

We used data from a previously published cross-sectional survey of IM CRs from 2018 (Garg M, et al., JAMA Netw Open 5:e223882, 2022).. As there is no publicly available national database of CRs, it was not possible to calculate a total number of eligible participants or select a random sample. Therefore, we employed snowball sampling as well as the Association of Program Directors in Internal Medicine listserv. Data were analyzed using descriptive statistics. We used Chi square testing to compare responses across gender, race/ethnicity, and academic versus community programs.

**Results:**

There were 169 respondents. Response rates were 57% in the snowball sample and 12% for the listserv. Forty-two percent of IM CRs were selected via formal application, compared to 49% selected via informal processes. Chi square testing showed a significant difference in formal versus informal selection by race/ethnicity; Black/African American CRs were more likely to be selected via formal processes compared to white, Hispanic/Latinx, and Asian respondents (*p* = 0.047). There were 69% who reported receiving some formal job training, with no differences in role preparation by gender, race/ethnicity, or program type.

**Conclusions:**

Standardization of selection and job training across IM chief residencies could improve consistency in role preparation, performance, and CRs’ experiences as well as supporting equity and diversity in CR selection.

## Introduction

Internal medicine (IM) chief residencies are prestigious leadership positions that provide vital functions in the United States’ graduate medical education, including trainee education, administrative services, and clinical care [1, 2]. In addition to their contributions to residency programs, many chief residents (CRs) consider the position an opportunity to develop leadership skills and launch careers in academic medicine [1].

Despite chief residencies’ importance to graduate medical education, data on the characteristics of chief residency positions are scarce and existing studies show variation across programs in CR roles and responsibilities, pay and benefits, and selection processes [1–3]. Processes used for CR selection are a particularly important area needing further characterization, as program directors and leaders in graduate medical education address the limited representation among CRs of physicians holding identities that are underrepresented in medicine [2, 4]. Additionally, few studies have addressed the role preparation provided to CRs. This may represent an area for intervention to improve CRs’ experiences and execution of integral roles within residency programs.

In the setting of calls for national standardization and reimagination of the CR role [5], we provide an updated characterization of the selection and role preparation processes of IM chief residencies.

## Methods

We used data from a previously published cross-sectional survey of CRs conducted in 2018 [1]. As has been previously described, there is no publicly available database or listserv containing all CRs across IM programs nationally [1, 6]. Therefore, this survey was by necessity non-representative and employed purposive rather than random sampling, including snowball sampling of the authors’ professional networks and an email listserv from the Association of Program Directors in Internal Medicine (APDIM) [1]. The survey was created using themes identified via literature review including an existing published survey [3] in addition to the experiences of our survey’s creators as former IM CRs [1]. Validation methods included expert review of survey drafts and pilot cognitive testing of the survey with former IM CRs [1]. Survey questions addressed selection processes, whether job training or workshops were provided, and program support to attend the APDIM annual CR meeting. Responses were compared by type of program (academic vs. community), gender, and race/ethnicity using Chi square testing. This study was determined to be exempt by the University of California, San Francisco Institutional Review Board.

## Results

A total of 169 CRs responded to the survey. Response rates were 57% in the snowball sample (70 of 122 invited participants) and 12% for the APDIM listserv (99 of 842 invited participants). Demographics, as previously published, are presented in Table 1 [1]. Table 2 details responses to selection and role preparation questions. Seventy-one respondents (42%) reported being selected through a formal application, while 82 (49%) were selected by program leadership without a formal application. Another 15 (9%) reported being selected via specific self-described processes including nomination by program directors or peers followed by interviews, selection by program directors themselves, or voting by fellow residents. Authors MG and BJ reviewed all free text responses related to selection process and adjudicated whether they described a formal application or more informal processes as described in the survey question; those that did not fit clearly into either category were labeled “other” for data analysis. Chi square testing showed a statistically significant association between method of selection and race/ethnicity (Table 3). A higher percentage of Black/African American respondents (4 of 8, 50%) were selected by a formal process compared to respondents who identified as Asian (20 of 44, 46%), white (34 of 89, 38%), or Hispanic/Latinx (8 of 18, 44%) (*p* = 0.047). This association was not seen when respondents underrepresented in medicine (AA, Hispanic/Latinx, American Native/Pacific Islander) were combined and compared with white/Asian. There were no statistically significant differences by gender or program type. A majority of respondents, 69%, reported receiving some formal job training. Of those who received formal training, the most common role preparation method was attendance at a conference or external meeting. There were no differences in role preparation by gender, race/ethnicity, or program type.

**Table 1:** Characteristics of survey respondents as previously published by Garg et al. in *JAMA Network Open*

Characteristic	Respondents, No. (%); *N* = 169^a^
Type of program	
Academic or university-based	125 (74)
Community-based	40 (24)
Military or uniform health services-based	4 (2)
Gender	
Female	77 (46)
Male	92 (54)
Other	0 (0)
Race and ethnicity	
African American or Black	8 (5)
African American or Black and Hispanic or Latinx	1 (1)
American Indian or Alaska Native	2 (1)
Asian	44 (26)
White	89 (53)
Hispanic/Latinx	19 (11)
Prefer not to answer	6 (4)
Post-Graduate Year (PGY)	
PGY 3	25 (15)
PGY 4	135 (80)
PGY 5	8 (5)
PGY 6 or beyond	1 (1)
Fellowship plans	
Already accepted to fellowship	69 (41)
Planning to apply	33 (20)
Returning in between fellowship years to do my chief year	2 (1)
None	58 (34)
Undecided	7 (4)

^a^Percentages may not add up to 100 due to missing responses

**Table 2:** Chief resident selection and role preparation survey responses

*Selection for CR role*		
Survey question		Respondents, No. (%); *N*=169^a^
How were you selected to be a CR?		
I applied through a formal application		71 (42)
I was selected by my residency program leadership without a formal application		82 (49)
Other process		15 (9)
Peer nomination then selection by program director		4 (27)^b^
Peer nomination then interview		8 (53)^b^
Self-nomination then voting by residents		2 (13)^b^
Invited to interview by program		1 (7)^b^
*Role preparation*		
Do you have job training or workshops in preparation for chief year?		
Yes		117 (69)
Meeting/training with outgoing chiefs		107 (92)^c^
Written documentation of roles and expectations		74 (63)^c^
Internal faculty led training or workshop		41 (35)^c^
Attendance at conference or external meetings		111 (95)^c^
No		42 (25)
Did you attend the APDIM Chief Resident meeting?		
Yes		139 (82)
No		19 (11)
Did your program support your attendance at the APDIM Chief Resident meeting?		
Financial support		51 (30)
Financial support and service coverage		93 (55)
I’m not sure		4 (2)
Neither		9 (5)
How much overlap did you have with previous year CRs?		
Days		38 (23)
Weeks		51 (30)
Months		19 (11)
None		50 (30)

^a^Percentages may not add up to 100% due to missing responses

^b^Calculated as a percentage of the 15 respondents who answered, ‘Other process’ to “How were you selected to be a CR?”

^c^Calculated as a percentage of the 117 respondents who answered, ‘Yes’ to “Do you have job training or workshops in preparation for chief year?

**Table 3:** Selection process for chief residency by race

Selection process	Race (No., %)			
	**African American**	**Asian**	**Caucasian**	**Hispanic**/**Latinx**
Formal	4 (50)	20 (45)	34 (38)	8 (44)
Informal	2 (25)	16 (36)	50 (56)	10 (56)
Other	2 (25)	8 (18)	5 (6)	0 (0)
Total	8 (100)	44 (100)	89 (100)	18 (100)

Pearson Chi2: 12.7

*p* = 0.047

## Discussion

Nearly half of respondents were selected for IM chief residency without a formal process. It is notable that our unadjusted analysis indicated a statistically significantly higher percentage of Black/African American respondents were selected through a formal application process. We thought it was important to keep racial groups disaggregated in our analysis in acknowledgment of the different experiences reported across various identities that are underrepresented in medicine [7]. Our sample size is small and thus conclusions cannot be made from this finding; furthermore, without existing data on the demographics of CRs nationally we are unable to conclude whether our study population is a representative cross section of CRs, though the low number of underrepresented in medicine respondents reflects the well known disproportionate numbers of their workforce representation compared to their numbers in the United States population [8]. This observation is hypothesis generating rather than dispositive, but signals the possibility of bias in informal and non-standardized selection processes, which may contribute to the limited diversity of CRs nationally and which requires further study.

As described by Johnson and colleagues, CR selection is often based on methods with inherent bias, including interpersonal relationships with residency leaders or subjective assessments with descriptive language that favors white over Black applicants. This mirrors biased selection processes across academia, which may contribute to the lack of significant progress made in improving racial diversity in academic IM faculty and leadership [4]. Furthermore, a recent study found that the majority (56%) of IM CRs over a 20 year period remain in academic medicine [6], suggesting an opportunity to impact faculty diversity by increasing CR diversity.

In terms of role preparation, nearly a quarter of CRs reported receiving no formal job training or workshops. While CRs are selected due to standout qualities among their resident peers, additional development is generally understood to be beneficial for those in leadership roles, especially the unique CR role, which has been characterized as “middle management” for residency programs [9]. A 2009 survey of IM residency program directors noted that APDIM attendance was the primary method of formal training for CRs, though mentorship was also frequently reported as a means of CR training. We similarly found that of the nearly 70% who received formal training, the most common form of role preparation was attendance at external conferences, specifically APDIM [3], while a high percentage also noted training through written documentation of role expectations and meetings with outgoing chiefs. This suggests little change in training methods over the nearly 10-year period between 2009 and 2018. Though it is the main training opportunity for CRs, it has not been studied whether one-time attendance at an external conference is effective training for the multifaceted IM CR role.

Targeted leadership development may create opportunities for candidates that may be historically overlooked or not exemplar across all criteria, and may expand the pool for potential chief resident candidates. There exists an unrealized potential for formal longitudinal skill-building in leadership, mentorship, and teaching among CRs as many prepare for academic careers. Additional job training in these areas could also improve their ability to provide high-quality services to IM residency programs including skilled teaching and mentoring of medical students and residents. The Accreditation Council for Graduate Medical Education could have an impactful role in setting national standards for selection and training of chief residents.

Limitations of our study include the use of purposive sampling and a non-representative sample; however, as described in reference number 1, this method was chosen given the significant challenges in sampling this population as discussed above [1]. Our data was collected in 2018, and selection practices may have evolved since that time, limiting generalizability of our findings. A future exploratory survey could better define selection process questions and ask program leadership for their viewpoints on selection as well as CRs. Future studies could also provide wider samples of the national CR population across specialties and address aspects of CRs’ training and selection experiences in more detail. In particular, further research is needed to explore the impacts of bias in both formal and informal CR selection processes, as well as the experiences of CRs who identify as members of groups underrepresented in medicine.

## Conclusions

Our findings support that there is opportunity for standardization of the IM CR selection and training in the United States. Expanded, longitudinal skill building could enhance CRs’ experiences and preparation for teaching and leadership roles in academic medicine. Future studies could explore whether nationally consistent job training CRs could improve their performance in supporting the academic, patient care, and administrative functions of IM residencies. Additionally, more research is needed to determine whether selection processes with attention to areas prone to bias could improve equity and diversity in these important leadership positions [4].

## Data Availability

The datasets generated and/or analyzed during the current study are not publicly available due the professionally sensitive nature of the survey questions but are available from the corresponding author on reasonable request.
